# Curcumin, Inflammation, and Chronic Diseases: How Are They Linked?

**DOI:** 10.3390/molecules20059183

**Published:** 2015-05-20

**Authors:** Yan He, Yuan Yue, Xi Zheng, Kun Zhang, Shaohua Chen, Zhiyun Du

**Affiliations:** 1 Institute of Natural Medicine & Green Chemistry, School of Chemical Engineering and Light Industry, Guandong University of Technology, 232 Wai Huan West Road, Guangzhou Higher Education Mega Center, Guangzhou 510006, China; E-Mails: hxg_129@126.com (Y.H.); yueyuan00@126.com (Y.Y.); xizheng@pharmacy.rutgers.edu (X.Z.); kzhang@wyu.edu.cn (K.Z.); 2 Susan Lehman Cullman Laboratory for Cancer Research, Department of Chemical Biology, Ernest Mario School of Pharmacy, Rutgers, The State University of New Jersey, Piscataway, NJ 08854, USA; 3 Department of Otorhinolaryngology, Guangdong General Hospital & Guangdong Academy of Medical Sciences, Guangzhou 510030, China; E-Mail: csh_rl@163.com

**Keywords:** curcumin, antioxidant, inflammation, chronic diseases

## Abstract

It is extensively verified that continued oxidative stress and oxidative damage may lead to chronic inflammation, which in turn can mediate most chronic diseases including cancer, diabetes, cardiovascular, neurological, inflammatory bowel disease and pulmonary diseases. Curcumin, a yellow coloring agent extracted from turmeric, shows strong anti-oxidative and anti-inflammatory activities when used as a remedy for the prevention and treatment of chronic diseases. How oxidative stress activates inflammatory pathways leading to the progression of chronic diseases is the focus of this review. Thus, research to date suggests that chronic inflammation, oxidative stress, and most chronic diseases are closely linked, and the antioxidant properties of curcumin can play a key role in the prevention and treatment of chronic inflammation diseases.

## 1. Introduction

*Curcuma longa* (turmeric) is a curry spice and a traditional Chinese medicinal herb with a long history of use as a treatment for inflammatory conditions in China and Southeast Asia [1]. Turmeric constituents include three curcuminoids (curcumin, demethoxycurcumin and bisdemethoxycurcumin), volatile oils (natlantone, tumerone and zingiberone), proteins, sugars and resins. It controls inflammation, cell growth and apoptosis, being thus useful to prevent and treat some diseases thanks to its anti-oxidant, and anti-inflammatory activities and excellent safety profile, most of which are attributed to the presence of curcumin [2]. Curcumin has been shown to be a highly pleiotropic molecule interacting with numerous inflammatory molecular targets. *In vitro* and *in vivo* studies, especially clinical trials, indicate curcumin may be a potential therapeutic agent in many chronic diseases such as inflammatory bowel disease, arthritis, pancreatitis, chronic anterior uveitis, and cancers [3]. Owing to its valuable properties, almost 100 companies are currently providing various curcumin products in the form of drinks, tablets, capsules, creams, gels, nasal sprays, extracts and coloring agents for both edible and medical needs [4].

Inflammation is an adaptive physiological response induced by deleterious circumstances including infection and tissue injuries. Observational studies have revealed that inflammation is the product of complex series of responses triggered by the immune system. Inflammation also causes a wide range of physiological and pathological morbidities [5]. Extensive research has shown that inflammation is associated with alteration of signaling pathways, which results in increased levels of inflammatory markers, lipid peroxides and free radicals. It has also been hypothesized that inflammation plays a central role in the wound healing process and in combating infection. Two stages of inflammation exist—acute and chronic inflammation. Acute inflammation is an initial stage of inflammation (innate immunity) mediated through the activation of the immune system, which persists only for a short time and is usually beneficial for the host. If the inflammation lasts for a longer time, the second stage of inflammation (chronic inflammation) starts and may initialize various chronic diseases such as obesity, diabetes, arthritis, pancreatitis, cardiovascular, neurodegenerative and metabolic diseases, as well as certain types of cancer [6]. Oxidative stress and oxidative damage are involved in the pathophysiology of many chronic inflammatory and degenerative disorders, which is followed by a decrease in health status and increasing probability of chronic diseases such as cancer, atherosclerosis, Alzheimer’s disease, metabolic disorders and so on. They are likely caused by low grade inflammation driven by oxygen stress as indicated by the increase of pro-inflammatory cytokines such as IL-6, IL-1 and TNF-α, genes encoded by activation of nuclear factor kappa-B (NF-κB) [7].

Curcumin shows strong anti-oxidation and anti-inflammatory activities. In the past two decades over 7000 articles have discussed the molecular basis of curcumin’s attributed antioxidant, anti-inflammatory, antibacterial, antiapoptosis, anticancer and related activities. Over 100 clinical trials have focused on the role of curcumin in various chronic diseases, including diabetes and cancers, as well as autoimmune, cardiovascular, neurological and psychological diseases [8]. In this review we try to clarify the possible link between curcumin, inflammation and chronic diseases.

## 2. Anti-inflammatory Mechanisms of Curcumin

Extensive research has demonstrated the mechanism by which persistent oxidative stress can lead to chronic inflammation, which in turn could cause many chronic diseases including cardiovascular diseases, neurological diseases, pulmonary diseases, diabetes and cancers [9]. Oxidative stress is defined as a disturbance in the balance between the production of reactive oxygen species (free radicals and reactive metabolites) and antioxidant defenses as their elimination by protective mechanisms. This imbalance causes the damage of important biomolecules and cells, as well as potential impacts on the organisms [10]. ROS play a central role both upstream and downstream of NF-κB and TNF-α pathways, which are located at the center of the inflammatory response. The hydroxyl radical is the most harmful of all the ROS. A schematic representation indicates the three loops involved in amplification of inflammation where loop 1 demonstrates the NF-κB-TNF-α positive feedback loop and loop 2 shows the redox sensing loop by ROS-NF-κB-TNF-α. Both loops can be blocked by using antioxidant like H2 that scavenges hydroxyl radicals directly or via NF-κB pathways. ROS are generated by Nox system and amplified through these loops. In addition, the modified proteins by ROS may generate a loop 3 which may promote the autoimmune response by feeding back into loops 1 and 2 [11,12].

Nuclear factor erythroid-2 related factor 2 (Nrf2) is highly related to oxidative stress in inflammation [13]. The role of Nrf2 has been addressed in kidney and heart in a model of chronic renal injury as well as in models of neuronal damage induced by quinolinic acid and in cerebellar granule neurons in culture [14,15,16,17]. There are also notably reports showing reciprocal regulation of Nrf2 and NF-κB, suggesting an anti-inflammatory role of Nrf2 and a large number of documents reported that Nrf2 is associated with MAPK, NF-κB, PI3K and PKC pathways [18,19]. Thus, Nrf may play an important role in pathologic study of multi-organ protector against oxidative damages [20]. Furthermore, evidence also suggested that mitochondrial dysfunction is a significant pathological mechanism in neurodegenerative diseases, renal damage, obesity, diabetes, liver and lung injuries [21].

**Figure 1:**
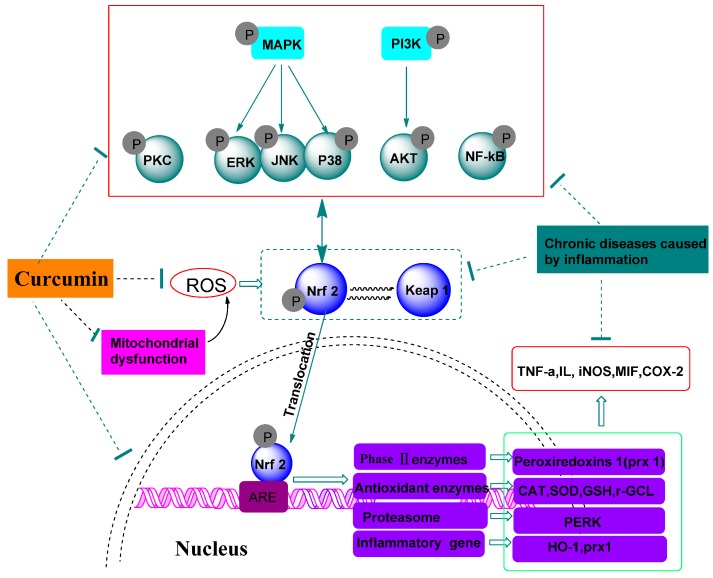
Inflammatory targets modulated by curcumin.

**Figure 2:**
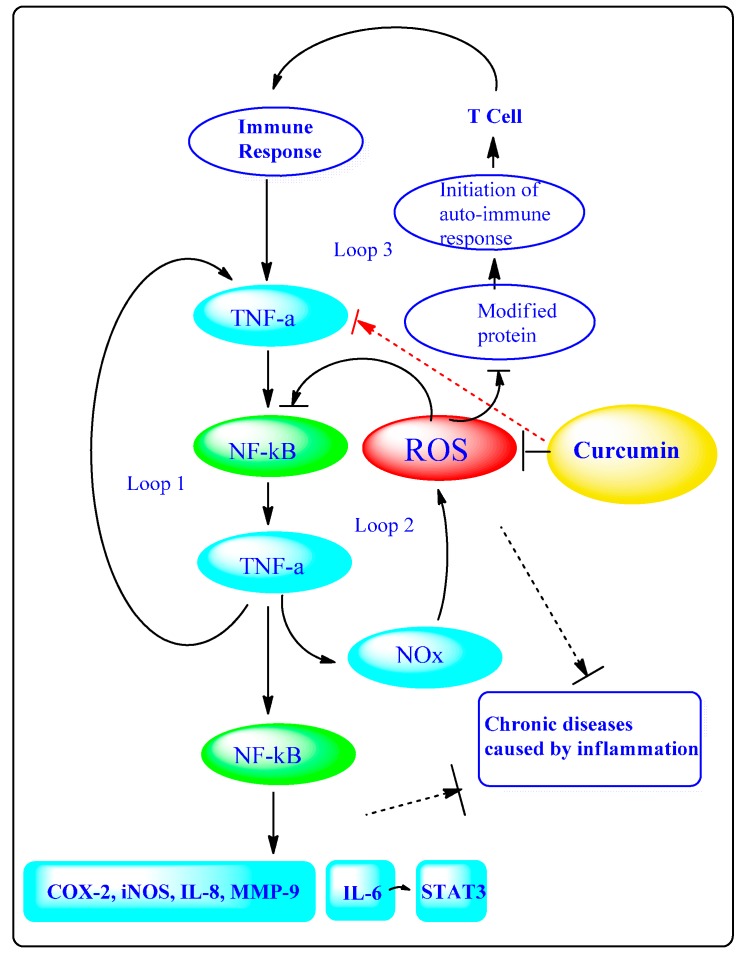
Relationship among ROS, chronic inflammation diseases and the antioxidative properties of curcumin.

Numerous mechanisms by which curcumin can display anti-inflammatory activity have been proposed (Figure 1 and Figure 2). It was suggested that curcumin alleviates oxidative stress, inflammation in chronic diseases through the Nrf2-keap1 pathway. Curcumin can suppress pro-inflammatory pathways related with most chronic diseases and block both the production of TNF and the cell signaling mediated by TNF in various types of cells. Curcumin may also be a TNF blocker from *in vitro* and *in vivo* studies by binding to TNF directly [22,23,24].

Due to its chemical structure, curcumin may act as a natural free radical scavenger. Curcumin can decrease the release of different interleukins through NF-κB. Curcumin could act as a stress response mimetic that induces some components of the protein homeostasis network or as it is known to bind amyloid, directly acts in the misfolded cascade [25]. The antioxidant activity and the free radical reactions of curcumin are closely related to its phenolic O-H and the C-H. It was found that the antioxidant mechanism of curcumin was based on the H-atom abstraction from the phenolic group, not on the central CH_2_ group in the heptadienone link. Curcumin, methylcurcumin, and half-curcumin with similar structure of O-H BDEs, indicated that the two phenolic groups were independent of each other [26,27].

## 3. Curcumin in Inflammation Induced Chronic Diseases

**Figure 3:**
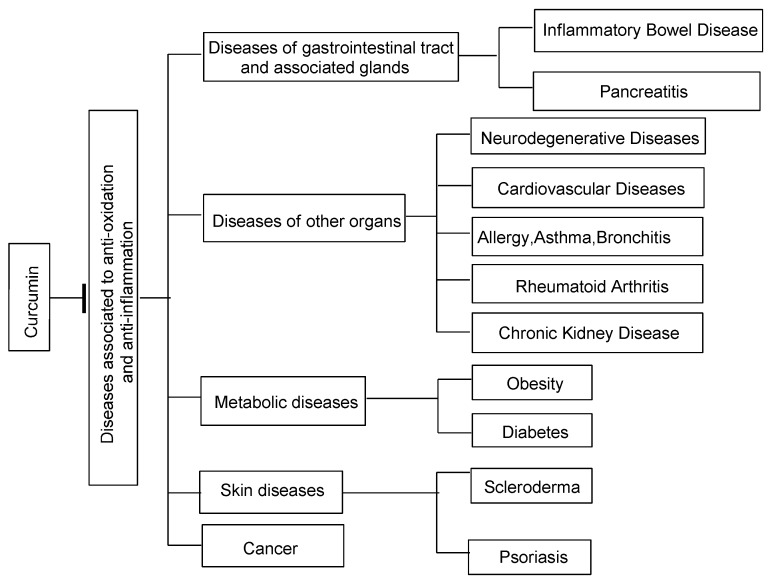
The main chronic diseases curcumin is active against.

Curcumin has been used as a remedy for the prevention and treatment of many organ and tissue disorders, most of which are associated with inflammation and oxidative stress. Curcumin alleviates oxidative stress, inflammation in chronic diseases and regulates inflammatory and pro-inflammatory pathways related with most chronic diseases (Figure 3).

### 3.1. Diseases of the Gastrointestinal Tract and Associated Glands

#### 3.1.1. Inflammatory Bowel Disease

Inflammatory bowel disease (IBD) is a chronic relapsing inflammation disease characterized by oxidative and nitrosative stress, leucocyte infiltration and up-regulation of proinflammatory cytokines. NF-κB is a key target for numerous IBD therapies, which is involved in the production of cytokines and chemokines integral for inflammation [28].

Many studies have been conducted to evaluate curcumin’s potential in patients with IBD for its efficacy as an anti-inflammatory without significant side effects [29,30,31,32]. McCann *et al.*, found different turmeric extracts could benefit the variants of SLC22A4 and IL-10 associated with IBD, by reducing inappropriate epithelial cell transport (SLC22A4, 503F) and increasing anti-inflammatory cytokine gene promoter activity (IL-10, -1082A) [33]. Beloqui *et al.*, designed a local delivery of curcumin using pH-sensitive polymeric nanoparticles and found it significantly decreased neutrophil infiltration and TNF-α secretion [34]. Curcumin is considered as an orally bioavailable blocker of TNF and other pro-inflammatory biomarkers [35].

Topcu evaluated the effects of curcumin on epithelial cell apoptosis, the immunoreactivity of the phospho-c-Jun *N*-terminal kinase (JNK) and phospho-p38 mitogen-activated protein kinases (MAPKs) in inflamed colon mucosa, and oxidative stress in a rat model of ulcerative colitis induced by acetic acid. Curcumin (100 mg/kg per day, intragastrically) was administered 10 days before the induction of colitis and was continued for two additional days. Curcumin treatments were associated with amelioration of macroscopic and microscopic colitis sores, decreased MPO activity, and decreased MDA levels in acetic acid-induced colitis rats. Oral supplementation of curcumin obviously prevented programmed cell death and restored immunreactivity of MAPKs in the colons. The results of this study suggest that oral curcumin treatment decreases colon injury and is associated with decreased inflammatory reactions, lipid peroxidation, apoptotic cell death, and modulating p38- and JNK-MAPK pathways [36].

Larmonier *et al.*, found that curcumin attenuated lipopolysaccharide (LPS)-stimulated expression and secretion of macrophage inflammatory protein (MIP)-2, IL-1β, keratinocyte chemoattractant (KC), and MIP-1α in colonic epithelial cells (CECs) and in macrophages. Curcumin significantly inhibited PMN chemotaxis against MIP-2, KC, or against conditioned media from LPS-treated macrophages or CEC, a well as the IL-8-mediated chemotaxis of human neutrophils. Curcumin inhibited random neutrophil migration with no toxic effects, suggesting a direct effect on neutrophil chemokinesis. Curcumin inhibited PMN motility by the downregulation of PI3K activity, AKT phosphorylation, and F-actin polymerization [37]. Epstein also demonstrated reduced p38 MAPK activation and IL-1β, enhanced IL-10 and dose-dependent suppression of MMP-3 in CMF in curcumin-treated mucosal biopsies [38]. Curcumin has been shown to attenuate colitis in the dinitrobenzenesulfonic acid (DNB)-induced murine model of colitis with a reduction in MPO activity, IL-1β expression, and reduction of p38 MAPK. Binion *et al.*, found curcumin may inhibit VEGF-mediated angiogenesis in human intestinal microvascular endothelial cells via down regulation of the COX-2 and MAPK [39,40]. Curcumin also inhibited the expression of VCAM-1 in HIMECs through the block of p38 MAPK, Akt, and NF-κB. Thus curcumin may represent a novel therapeutic agent targeting endothelial activation in IBD [41,42]. Curcumin showed a protective effects on 2,4,6-trinitrobenzenesulphonic acid-induced colitis in mice. Curcumin also reduced NO and O_2_ levels, which were associated with the effective expression of Th1 and Th2 cytokines and inducible NO synthase. NF-κB activation in colonic mucosa was also suppressed in the curcumin-treated mice [43].

#### 3.1.2. Pancreatitis

Chronic pancreatitis (CP) is associated with progressive fibrosis, pain and/or loss of exocrine and endocrine functions, of which pain is the main symptom [44]. The key etiological factors in CP are alcohol and tobacco abuse, genetic, environmental, hypertriglyceridemia, hypercalcemia, autoimmune and sometimes idiopathic [45]. Alcohol and its metabolites could produce oxidative stress, regulate a series of oxidant-related factors and eventually result in chronic pancreatitis. They regulate the NF-κB, activator protein-1 (AP-1) in acinar cells and three classes of MAP kinases, which were inhibited by antioxidants [46,47]. Alcohol metabolism also produces free radical and induces the CYP450 enzymes resulting in bioactivation [48]. The pathologies of pancreatitis are difficult to clearly define [49]. Recently progresses in chronic pancreatitis mainly concern the early diagnosis of the disease, the prediction of the fibrosis degree of the gland, the medical and surgical treatment of abdominal pain and the knowledge of the natural history of the autoimmune pancreatitis [50].

In recent years, it has been shown that curcumin has a highly pleiotropic molecule capable to contact numerous molecular targets with pancreatitis [51]. In view of early cell culture and animal model research methods, clinical trials reveal curcumin may be therapeutic candidate in pancreatitis [52]. In the rats model of induced pancreatitis, curcumin reduced inflammation by dramatically decreasing activation of NF-κB and AP-1 as well as suppressing mRNA induction of iNOS, TNF-a, and IL-6 in the pancreas [53]. In addition, curcumin acted on inflammatory mediators to improve disease’s severity as measured by histology, serum amylase, pancreatic trypsin, and neutrophil infiltration in both ethanol- and cerulein- induced pancreatitis [54]. In one clinical study, 25 patients, aged 43–77 years old, were needed to consider the biological activity and safety of curcumin in pancreatic cancer patients by oral administration with 8 g of curcumin capsules, the down-regulation of NF-κB and COX-2 suggested curcumin was effective enough in pancreatic cancer [55]. Another pilot study was undertaken to investigate the clinical efficacy of oral curcumin (500 mg) with piperine (5 mg) on the pain and the markers of oxidative stress in patient with pancreatitis, and showed that this oral administration regime was able to suppress the lipid peroxidation in patients who had pancreatitis following with downgrade of the levels of malonyldialdehyde (MDA) and glutathione (GSH) in red blood cell [56].

### 3.2. Diseases of Other Organs

#### 3.2.1. Neurodegenerative Diseases

Neurodegenerative diseases may affect millions of people yearly and the incidence is increasing as the population ages. About one in five Americans over the age of 65 will be diagnosed with a neurodegenerative disease by 2030 as shown by the NIH [57]. Over the last several decades a broad range of studies have demonstrated the progression of age-dependent neurodegeneration is associated with decreased antioxidants and increased oxidative damage to proteins, DNA and lipids [58,59]. Modification of oxidative protein occurs at a persistent low level in diverse cells and tissues, and accumulates in neurodegenerative diseases [60].

The considerable excitement about curcumin’s preclinical efficacy for neurodegenerative diseases mainly focused on its lack of toxicity and low cost. Kim *et al.*, summarized that curcuminoids possess diverse biological properties that modulate debilitating biochemical processes involved in Alzheimer’s diseases, that include attenuation of mitochondrial dysfunction-induced oxidative stress and inflammatory responses to inflammatory cytokines, COX-2, and nitric oxide synthase (iNOS), in addition to neurodamage caused by heavy metal poisoning [61]. Banji *et al.*, observed the expression of histological assessment of the CA1 region of the hippocampus, caspase-3 and cleaved caspase-3, showing curcumin can effectively reduce the levels of proteins, cleaved caspase-3 and mitochondrial enzymes to protect the brain [62]. Thus mitochondrial dysfunction plays an important role in pathogenesis of neurodegenerative diseases including AD [63]. Curcumin treatment was found to repress the gene transcription of early growth response gene-1 (Egr-1), which mediates TNF-a, IL-1β, IL-8, MIP-1β, and MCP-1 in PBM and THP-1 cells through the interaction of amyloid-b-proteins (Ab). In the AD transgenic Tg2576 mouse brain, curcumin significantly lowered the levels of oxidized proteins and IL-1β, and decreased the levels of insoluble and soluble Ab and plaque burden without affecting amyloid precursor protein. Curcumin has been evaluated in a clinical trial for the prevention of AD [64,65].

#### 3.2.2. Cardiovascular Diseases

Cardiovascular Diseases (CVDs), including heart disease, vascular disease and atherosclerosis, are the most critical current global health threat. Epidemiological and clinical trials have shown strongly consistent relationships between the inflammation markers and risk of cardiovascular diseases [66]. It is widely appreciated that the key mechanisms in the development of CVDs are inflammation and oxidant stress, activation of pro-inflammatory cytokines, chronic transmural inflammation and C reactive protein (CRP) [67]. Thus cytokines, other bioactive molecules, and cells that are characteristic of inflammation are believed to be involved in atherogenesis.

Abundant evidence suggests that curcumin mediates its effects against CVDs through diverse mechanisms such as oxidative stress, inflammation and cell death [67,68,69,70]. Curcumin was able to protect against inflammation, cardiac hypertrophy and fibrosis by the inhibition of p300-HAT activity and downstream NF-κB, GATA4 and other signal pathways. Curcumin suppressed lipopolysaccharide (LPS)-induced overexpression of inflammatory mediators in vascular smooth muscle cells (VSMCs) of rats via inhibition of the TLR4-MAPK/NF-κB pathways, partly due to block of NADPH-mediated intracellular ROS production [71]. LPS not only dramatically increased expression of inflammatory cytokines (MCP-1, TNF-α, TLR4 and iNOS) and NO production, but also significantly increased phosphorylation of IκBα, nuclear translocation of NF-κB (p65) and phosphorylation of MAPKs in VSMCs. Furthermore, LPS significantly increased production of intracellular ROS, and decreased expression of p47 (phox) subunit of NADPH oxidase. Curcumin concentration-dependently attenuated all the aberrant changes in LPS-treated VSMCs [72]. Parodi *et al.*, demonstrated that curcumin-treated mice exhibited relative decreases in aortic tissue activator protein-1 and NF-κB DNA binding activities and significant lower concentrations of IL-1β, IL-6, MCP-1, and MMP-9 in experimental AAAs [73]. Curcumin may affect signal transduction (e.g., Akt, AMPK) and modulate specific transcription factors (such as SREBP1/2, NRF2, FOXO1/3a, CREBH, CREB, PPARγ, and LXRα) which regulate the expression of genes in free radicals scavenging (MnSOD, catalase, and heme oxygenase-1) and lipid homeostasis (CD36, aP2/FABP4, HMG-CoA reductase, and CPT-1). Curcumin could induce mild oxidative and lipid-metabolic stresses, which lead to an adaptive cellular stress response, by stimulating the cellular antioxidant defense systems and lipid metabolic enzymes [74]. Duan *et al.*, indicated the post-treatment of curcumin have an effects against myocardial ischemia and reperfusion by the activation of JAK2/STAT3 pathway, which reflected by the annulment of the curcumin-induced down-regulation of Caspase3 and up-regulation of Bcl2 [75]. Curcumin was also found to be a novel heart failure therapy by the GATA4/p300 transcriptional signal pathway which is recognized as a critical role in the cardiomyocyte hypertrophy and heart failure therapy [76]. Also, curcumin may inhibit PI3K/Akt/NF-κB signaling pathway, reduce the inflammatory response, and thus provide a protective effect against CVB3-induced myocarditis [77]. Curcumin was found to stimulate the apoptotic cell death of H9c2 cells by upregulating ROS generation and triggering activation of JNKs [78]. Interestingly, curcumin exerts a pro-oxidative activity, with 2′,7′-dichlorofluorescin diacetate (DCFH-DA) staining revealing up-regulation of ROS levels and anti-oxidants found to abrogate PARP cleavage.

#### 3.2.3. Allergy, Asthma and Bronchitis

The initiation and maintenance of asthma and allergy and bronchitis underlies the inflammation pathways relevant to the perplexing rise of these chronic inflammatory disorders. That allergy, a proinflammatory disease, is normally mediated through inflammatory cytokines, such as T helper-2 CD4 T (Th2) cells and Th2-associated cytokines, as well as IL-17-associated neutrophilic airway inflammation [79]. Asthma is also an inflammatory disease in which eotaxin, MCP-1 and MCP-3 play a crucial role [80]. Eosinophils are key cells of allergic inflammation and their adhesion onto human bronchial epithelial cells is mediated by a CD18-intracellular adhesion molecule (ICAM)-1-dependent interaction.

As shown in *in vivo* and *in vitro* experiments, curcumin can help clear constricted airways and increase antioxidant levels. Curcumin was reported to have a major role in reducing the allergic response using a murine model of allergy [81]. Nilani *et al.*, studied selected plants extracts with anti-asthmatic constituents. The results showed that curcumin could be utilized in alternate anti-asthmatic therapy for they play a vital role in scavenging nitric oxide (NO) which could prevent the bronchial inflammation in asthmatic patients [82]. Rennolds *et al.*, determined that two distinct pathways controlled secretion of IL-6 and IL-8 where the cadmium-induced IL-6 secretion occurs via a NF-κB pathway and the IL-8 secretion involves the Erk1/2 signaling pathway [83]. The natural antioxidant curcumin could prevent both secretions by human airway epithelial cells. Ammar *et al.*, designed a study for the inhibitory effects of curcumin on the asthma related biological changes and studied the effects on serum IgE and the changes in the mRNA levels of iNOS, TNF-α and TGF-β1. Serum IgE was significantly decreased by curcumin. Curcumin was more potent in inhibiting mRNA expression of TNF-α [84]. Curcumin clearly attenuates allergic airway inflammation by inhibition of NF-κB and its downstream transcription factor GATA3. Chong *et al.*, investigated the anti-inflammatory effect of curcumin on acute allergic asthma in BALB/c mice. Notch1 and Notch2 receptor, especial Notch1 receptor, were found to be important in the development of allergic airway inflammation. Curcumin-treatment improved the airway inflammatory cells infiltration and down regulated the levels of Notch1/2 receptors and GATA3. The inhibition of Notch1-GATA3 signaling pathway by curcumin can prevent the development and deterioration of the allergic airway inflammation [85]. Thakare *et al.*, found curcumin could prevent significantly elevation of eosinophil peroxidase in nasal homogenate and serum IgE, NO, IL-4 in nasal lavage with an ovalbumin induced allergic rhinitis in guinea pig model [86]. Curcumin markedly attenuated allergic airway inflammation in asthma model by regulating Treg/Th17 balance where obvious inhibition of Th17 cells and significant increase of Treg cells were observed [87]. These findings support the possible use of curcumin as a therapeutic drug for patients with allergic asthma. Chung *et al.*, found curcumin administration markedly suppressed IgE-mediated and eosinophil-dependent conjunctival inflammation with less IL-4 and IL-5 (Th2 type cytokine) production in conjunctiva, spleen and cervical lymph nodes in the curcumin-administered mice [88]. OVA challenge stimulated the activation of the production of iNOS and curcumin treatment inhibited iNOS production in the conjunctiva. Curcumin also has wide pharmacokinetic effects as an inhibitor of NF-κB, eIF-2α dephosphorylation, proteasome and COX2 [89].

#### 3.2.4. Rheumatoid Arthritis

Rheumatoid arthritis (RA) could give rise to a systemic chronic inflammatory disorder and may impact many organs and tissues but mainly attack flexible (synovial) joints [90]. It was reported that oxidative stress made an important contribution to joint destruction in RA [91,92,93]. ROS is a significant mediator that activates a variety of transcription factors including NF-κB and AP-1, thus regulating the expression of over 500 different genes, such as growth factors, chemokines, cell cycle regulatory molecules, inflammatory cytokines and anti-inflammatory molecules [94]. Therefore, transcription factors and genes, involved in inflammation and anti-oxidation, are suspected to play a crucial adjective function in RA.

The main treatment of RA is to reduce arthritis reaction, inhibit disease development and irreversible bone destruction, protect the joints and muscle function, and ultimately achieve complete remission or low disease activity. Treatment principles include patient education, early treatment and combination therapy [95]. Drug therapy includes non-steroidal anti-inflammatory drugs (NSAIDs), slow-acting antirheumatic drugs, immunosuppressive agents, immune and biological agents and botanicals. NSAIDs are most common [96]. Curcumin is one of the NSAIDs with anti-inflammatory and anti-oxidant actions both *in vivo* and *in vitro* [97]. Many studies with animal and cells have elucidated the biological effects and molecular mechanisms of curcumin. A few clinical trials are underway now. Curcumin has raised interest as an agent of potential use in therapy of RA with the regulatory function of the related inflammatory factors associated with anti-oxidation [98]. Curcumin treatment activated caspase-3 and -9, up-regulated Bax, down-regulated Bcl-2 and Bcl-xL, and degraded poly (ADP-ribose) polymerase (PARP) with dose-dependent in the synovial fibroblasts from a previous study on patients with RA [99]. There also presented an inflammatory response in synovial fibroblasts by suppression of COX-2 after inhibition of prostaglandin E2 synthesis accompanied by curcumin [100]. Lee *et al.*, studied the effects of a curcumin-like diarylpentanoid [2,6-bis(2,5-dimethoxybenzylidene)cyclohexanone] in cellular targets of rheumatoid arthritis *in vitro* and demonstrated the compound abolished the p65 NF-κB nuclear translocation as well as binding activity of NF-κB DNA in the PMA-stimulated synovial fibroblasts via inhibited COX-2, IL-6, MMP-3, collagenase and pro-gelatinase B(pro-MMP-9) [101]. Curcumin inhibited AKT and IL-1β-induced NF-κB activation on account of degradation correlated with down-regulation of COX-2 and MMP-9 and reducing IκBα phosphorylation in IL-1β- and TNF-α- stimulated human articular chondrocytes. It showed the similar results when curcumin was analyzed in TNF-α-stimulated articular chondrocytes [102]. In another study, curcumin (500 mg) and diclofenac sodium (50 mg), alone or together, were administered to three groups of patients with RA. Curcumin may be the RA therapy candidate with the best improvement in the overall Disease Activity Score and American College of Rheumatology scores (tests used in clinical practice and clinical trials to evaluate symptoms of RA and disease progression) of all three groups [103].

#### 3.2.5. Chronic Kidney Diseases

Chronic kidney disease (CKD), an inflammatory disease, is defined by either a progressive atrophy of glomerular filtration rate (GFR) and/or the presence of abnormalities in the urine such as white blood cells, protein and red blood cells [104,105]. Two main causes of CKD can be attributed to hypertension and diabetes mellitus (DM) which major pathological and are end-stage interstitial fibrosis, glomerular hypertrophy and sclerosis, accumulation of extracellular matrix (ECM) in the glomerular basement membrane and mesangial cell proliferation [106,107]. Since biological markers of oxidative stress are markedly elevated in CKD patients, oxidative stress gains concern as a contributing factor to CKD pathology [108]. Nrf2 regulates the expression of a wide array of thiol molecules and their generating enzymes, detoxifying enzymes, genes encoding antioxidant proteins and stress response proteins [109].

Currently, there is no definite treatment to improve kidney function in CKD. It has been well document that curcumin could disrupt the Nrf2-Keap1 complex with upregulation of the activity and expression of HO-1 in renal cells as a consequence to protect the kidney functions [110]. There is considerable evidence suggesting that Nrf2 signaling plays a protective role in renal injuries [111,112]. In addition, impaired Nrf2 consequent target gene repression and activity have been observed in CKD animals [107]. Therefore, a pharmacological intervention activating Nrf2 signaling can be benefit for protecting against kidney dysfunction in CKD [113]. Moreover, curcumin treatment has been shown to decrease macrophage infiltration in the kidneys of chronic renal failure rats and to block transactivation of NF-κB, indicating that the anti-inflammatory property of curcumin may be responsible for alleviating disease in this animal model [114]. In addition to the above reports, Waly *et al.*, reported that curcumin significantly ameliorates oxidative stress by reduction the levels of TAC and GSH as well as inhibition of the activities of CAT, GPX enzymes and SOD in human embryonic kidney (HEK) 293 cells [115]. Gaedeke *et al.*, found that curcumin blocks TGF-β-induced expression of several mediators of fibrosis by inhibition of the transcription factor c-jun/AP-1, or through down-regulation of TβRII expression [116]. Siddhartha *et al.*, summarized that curcumin can blunt and/or strengthen the action and generation of some inflammatory mediators playing a role in CKD, such as eicosanoids, cytokines, reactive oxygen species (ROS), growth factors and transcription factors, thus showing potential anti-inflammatory effects in CKD [104]. Jane *et al.*, showed that curcumin could inhibit p300 and NF-κB actions and decrease oxidative stress through down-regulation of vasoactive factors (endothelial nitric oxide synthase and enothelin-1), transforming growth factor-β and extracellular matrix proteins in the kidneys with real-time reverse transcriptase polymerase chain reaction analyses [117]. In one animal experiment, Sprague-Dawley rats were subjected to 5/6 nephrectomy and randomly assigned to untreated (Nx), sham-operated rats served as controls, telmisartan-treated groups (10 mg/kg/day, orally; as positive control) curcumin-treated (75 mg/kg/day, orally). This research showed curcumin and telmisartan treatment can decrease creatinine clearance. The Nx rats demonstrated reduced Nrf2 protein expression. Moreover, curcumin had been reported that it ameliorated NF-κB p65, nicotinamide adenine dinucleotide phosphatase oxidase subunit (p67phox and p22phox), TGF-β1, cyclooxygenase-2, TNF-α and fibronectin accumulation to lower glutathione peroxidase activity and higher kidney malondialdehyde concentration in remnant kidney in Nx animals [118]. On the other animal research, the authors evaluated the link of renal, mitochondrial function and oxidant stress through K_2_Cr_2_O_7_-induced schemes, and revealed the therapeutic effect of curcumin on oxidant stress, renal dysfunction, histological damage and antioxidant enzyme activity both in kidney tissue and in mitochondria [119].

### 3.3. Metabolic Diseases

#### 3.3.1. Diabetes

Type 2 diabetes is a chronic disease where cells have reduced insulin signaling, leading to hyperglycemia and long-term complications, such as heart, kidney and liver disease. Recently, more and more studies have shown the critical roles of oxidative stress and inflammatory reactions in the pathogenesis of diabetes. When macrophages are activated by dying or stressed cells, the transcription factor NF-κB is induced and thus leads to the production of pro-inflammatory cytokines including TNF and IL-6.

**Figure 4:**
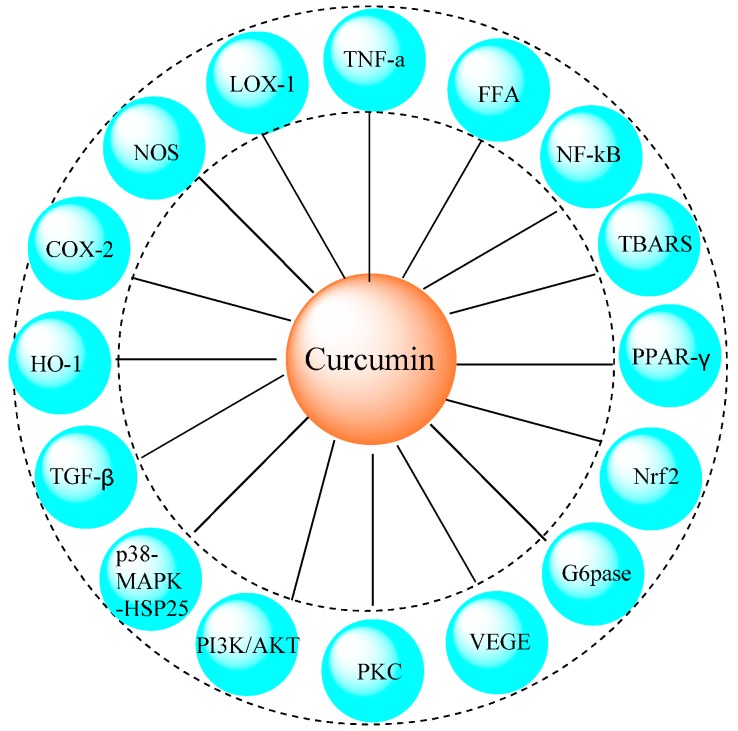
The anti-oxidative and anti-inflammatory molecular targets of diabetes for curcumin [126].

Curcumin is an anti-oxidant and NF-κB inhibitor and can be considered helpful for the prevention and amelioration of diabetes [120,121]. It is determined that curcumin can inhibit the enzymes linked to diabetes such as a-glucosidase, aldose reductase and aldose reductase inhibitors [122,123,124]. Aldebasi *et al.*, reported that curcumin has a therapeutic potential in the inhibition or slowing down progression of diabetic retinopathy through antioxidant, anti-inflammatory, inhibition of vascular endothelial growth and nuclear transcription factors [125]. Zhang *et al.*, summarized the recent applications of curcumin for the glycemia and diabetes-related liver disorders, neuropathy, adipocyte dysfunction, vascular diseases, nephropathy and pancreatic disorders. They also discussed its antioxidant and anti-inflammatory properties (Figure 4).

#### 3.3.2. Obesity

Numerous researchers has revealed that obesity is a proinflammatory disease, which is a major risk for atherosclerosis, cancer, type 2 diabetes, and other chronic diseases. Curcumin exhibits its activity against obesity by anti-inflammatory and antioxidant mechanisms. Curcumin as a treatment for obesity and obesity-related metabolic diseases has been shown extensively through suppressing the proinflammatory NF-κB, signal transducer and activators of STAT3, and Wnt/β-catenin. It activates peroxisome proliferator-activated receptor-gamma and Nrf2 cell signaling pathways, which could lead to not only the down-regulation of adipokines, including tumor necrosis factor, IL-6, leptin, resistin and monocyte chemotactic protein-1, but also the up-regulation of adiponectin and other gene products [127]. Mangge *et al.*, reported that curcumin can suppress the level of leptin release and chronic immune-mediated inflammation through its antioxidant to relieve the obese state [128]. Bradford *et al.*, showed the experimental evidence for the activity of curcumin in promoting the weight loss and reducing the incidence of obesity-related diseases [129]. Administration of Meriva (curcumin and phosphatidylcholine) was provided for at least 4 weeks and was found to be helpful for patients with diabetic microangiopathy and retinopathy at a dose of two tablets/day (corresponding to 100 mg of curcumin) [130]. Further study in diabetic patients also revealed that curcumin lowers the atherogenic risks by reducing the insulin resistance, triglyceride, uric acid, visceral fat and total body fat. Curcumin also helps to improve the relevant metabolic profiles in type 2 diabetic population [131,132]. And another clinical trial for pilot study of curcumin for women with obesity and high risk for breast cancer is in recruiting [133].

### 3.4. Skin Diseases

#### 3.4.1. Scleroderma

Scleroderma is a kind of connective tissue disease, typically resulting in vasculopathy and fibrosis of skin and other organs, [134]. The cause of scleroderma is still not clear, but fibrosis, vascular abnormalities and increased extracellular matrix production may be the causes [135]. Recently, oxidative stress plays an important role in the development of disease [136]. Several studies have verified the increased content of free radicals such as hydroxyl and peroxynitrite radicals, and increased serum levels of 8-isoprostane, a marker of oxidative stress in patients with scleroderma in human patients with scleroderma [137]. Additionally, mice treated with releasing agents of free radicals show cutaneous fibrosis [138]. So, many skin diseases may be connected with oxidative stress, which leads to inflammatory diseases.

Immune-suppression has been considered to be an anchor treatment, since perivascular infiltrate of inflammatory cells and activation of the immune system are key features of scleroderma [139]. Excessive accumulation of extracellular matrix (ECM) is the hallmark of scleroderma and results in inflammation [140,141]. Inflammation can be initiated and propagated by ECM disruption in all tissues. Molecules of ECM, newly liberated by injury or inflammation, include hyaluronan fragments, tenascins and sulfated proteoglycans. These act as ‘damage-associated molecular patterns’ or ‘alarmins’ that trigger and subsequently amplify inflammation [138]. Curcumin possess the effects of the anti-fibrosis, which is characterized by the reduction of collagen deposition, ECM production in pulmonary fibrosis and keloid formation [142]. The two PKC isoforms (δ and ε) play an important role in scleroderma. Wermuth *et al.*, suggested that curcumin administration could down-regulate the levels of PKC δ that cause ECM excessive accumulation and fibrosis *in vivo* and *in vitro* [143]. There is abnormal regulation of inflammatory cytokines and NF-κB involved in angiogenesis and fibrosis in scleroderma [144]. Curcumin can induce apoptosis in scleroderma lung fibroblasts (SLF), by inducting GST P1 and HO-1 which involve the inhibition of protein kinase C epsilon (PKCε). Thus PKC epsilon and phase 2 detoxification enzymes provide protection against curcumin-induced apoptosis in SLF. Song observed that curcumin effectively inhibited the down-regulation of TGF-β- induced factor (TGIF) to modulate TGF-β cascade [145]. Another study suggested that curcumin may have therapeutic effect in the treatment of scleroderma for it could protect rats against lung fibrosis induced by a large number of agents [146]. In conclusion, curcumin has a potentially function in the treatment of scleroderma, but plentiful researches are also needed.

#### 3.4.2. Psoriasis

Skin diseases seriously affect people’s health wih common and multiple features, of which psoriasis is the most common [147,148]. Psoriasis is a chronic inflammatory skin disease characterized by thick, red and scaly lesions on any part of the body which affects approximately 2% of the population worldwide [149]. Recent investigations revealed that there was a great success to link oxidative stress and autoimmune skin diseases [150]. The skin is continually under attack by ROS from both exogenous and endogenous sources. Researchers have demonstrated that dermal γδ T cells play an important role in the disease development. Many cytokines, including interleukin-23(IL-23), IL-17A, TNF-α, IL-6, IL-1β and IL-22, are also involved in the pathogenesis of psoriasis [151].

Curcumin is well known for its protective properties in treating various disorders of skin diseases. Curcumin protects skin by reducing inflammation and quenching free radicals through modulating TGF-β, NF-κB and mitogen-activated protein kinase pathway. Curcumin also regulates the phase II detoxification enzymes which are crucial in detoxification reactions and oxidative stress [152]. There are strong scientific rational suggestions that curcumin is potential herb to suppress psoriasis by inhibiting keratinocyte proliferation [153]. Sun *et al.*, revealed that curcumin is capable of relieving TPA-induced skin inflammation by down-regulating IFN-γ production and TPA-induced Th1 inflammation in K14-VEGF transgenic mice [154]. Curcumin could also demonstrate the inhibitory effect in lmiquimod-induced psoriasis-like inflammation by decreasing the levels of IL-1β and IL-6 [155]. Furthermore, Kurd’s clinical trial suggested that orally administered curcumin had a therapeutic effect without adverse events in patients with psoriasis according to some endpoints like psoriasis area, severity index score and safety [156].

### 3.5. Cancer

Inflammation plays decisive roles in all the ways of tumorigenesis and therapy response [157,158]. Activation and interaction between STAT3 and NF-κB are very vital in the control of cancer cells and inflammatory cells [159,160]. TNF-α, VEGF, IL-10, MMP-2 and MMP-9, MCP, CD4+ T, AP-1, Akt, PPAR-γ, MAP kinases and mTORC1 are also important linking factors between inflammation and cancer [161,162].

Curcumin has been found to have clinical therapeutic and prevention potential for cancer patients in *in vitro* and *in vivo* animal and human clinical studies for colorectal, liver, pancreatic, lung, breast, uterine, ovarian, prostate, bladder, kidney, renal, brain, non-Hodgkin lymphoma and leukemia cancers [163,164,165,166,167]. Regular consumption of turmeric has been associated to lower cancer rates in India although without quantitative cause-and-effect relationship data [168,169]. Curcumin acted as a modulator of intracellular signaling pathways on multiple targets which control tumor growth, angiogenesis, metastasis, inflammation, invasion and apoptosis [170]. Most carcinogens activate NF-κB pathways which leads to the expression of inflammatory enzymes and mediators, including COX-2, LOX-2, iNOS, inflammatory cytokines, especially TNF-α and chemokines [171]. Curcumin has shown anti-proliferative effect and is an inhibitor of the transcription factor NF-κB and downstream gene products (including fas, p53, VEGF, Cdc42, Bcl-2, COX-2, NOS, cyclin D1, TNF-α, interleukins and MMP-9) [172,173,174,175,176,177]. Curcumin provided a possibility for multiple myeloma treatment by down-regulating activation of NF-ΚB and STAT3 and suppressing COX-2 expression [178]. Karunagaran *et al.*, revealed that curcumin-induced apoptosis mainly involves the mitochondria-mediated pathway in various cancer cells. Kronski *et al.*, showed that as a chemopreventive curcumin inhibits the expression of the proinflammatory cytokines CXCL1 and -2 leading to diminished formation of breast and prostate cancer metastases. MiR181b is induced by the chemopreventive curcumin and inhibits breast cancer metastasis via down-regulation of the inflammatory cytokines CXCL1 and -2 [179,180]. Thus curcumin could serve as a simple bridge to bring metastamir modulation into the clinic in preventive and therapeutic effects. Prusty and Das explored the redox regulatory pathway involved in the HPV expression which can be modulated by an antioxidant-induced reconstitution of the AP-1 transcription [181]. Later they observed curcumin completely down-regulated the AP-1 binding activity and reversed the c-fos/fra-1 transcription to a normal state in cervix HeLa cancer cells which was a novel mechanism controlling transcription of pathogenic HPVs during keratinocyte differentiation and progression of cervical cancer.

### 3.6. Bioavailability of Curcumin

Despite curcumin’s highly promising features for treatment and prevention of various diseases, clinical uses have been hindered by poor absorption, rapid metabolism, short biological half-life, and low oral bioavailability (only 1% in rats) [182,183]. Very high doses (>3.6 g/day in humans) are required to produce any medicinal effect [184].

Wahlstrom and Blennow first reported in 1978 that negligible amounts of curcumin were observed in blood plasma after oral administration of 1 g/kg of curcumin in Sprague-Dawley rats due to its poor absorption from the gut [185]. Curcumin bioavailability may also be poor in humans, as either undetectable or extremely low serum levels of curcumin (0.006 ± 0.005 µg/mL at 1 h) were observed in humans after an oral dose of 2 g/kg [186]. It has been found that 10 mg/kg of curcumin given intravenously in rats gave a maximum serum curcumin level of 0.36 µg/mL, whereas a 50-fold higher curcumin dose administered orally gave only 0.06 ± 0.01 µg/mL maximum serum level in rat [187]. Intravenous administration of 2 mg/kg curcumin to rats showed better availability with the concentration was 6.6 µg/mL of blood plasma shown by Sun *et al.* [188]. A number of studies have cited extremely low blood curcumin concentrations (Table 1), indicating that curcumin bioavailability needs to be improved to exert significant medical effects.

**Table 1:** Previously reported blood curcumin concentrations in humans.

Subject	Dose (g/Day)	Sample Size	Plasma Curcumin Level (Means ± SE)	Ref.
Healthy volunteers	2	8	6 ± 5 ng/mL	[186]
	8	6	0.6 μg/mL	[189]
	12	1	57.6 ng/mL (t = 2 h)	[190]
Persons with Alzheimer’s Disease	4	30	7.76 ± 3.23 ng/mL	[191]
Patients with precancerous lesions	8	2	1.77 ± 1.87 mM	[192]
Patients with chronic inflammatory bowel disease	4	4 2	Pre-intervention 7.3 ± 8.1 ng/mL Post- intervention 3.8 ± 1.3 ng/mL	[193]
Patients with pancreatic cancer	8	5	134 ± 70 ng/mL	[194]
Patients with colorectal cancer	3.6	4	12.7 ± 5.7 nmol/g (normal tissue) 7.7 ± 1.8 nmol/g (malignant colorectal tissue)	[195]

Despite its generally low bioavailability, curcumin has been shown to have distant or indirect effects [104] through the upregulation of the enzyme intestinal alkaline phopshatase (IAP), which is a fantastic anti-oxidant and anti-inflammatory endogenous component produced at the gut epithelial level and that has been shown to have local and also distant protective effects though oxidation and inflammation down-regulation [196].

To improve the bioavailability of curcumin, numerous attempts have been made for challenges through the use of adjuvants like piperine [197], curcumin structural analogues [198], and development of improved delivery technologies such as nanodisks [199], polymeric micelles [200], nanoparticles [201] and polymeric implants [202].Combination therapy containing curcumin and a bio-enhancer such as piperine, quercetin or silibinin could enhance the cellular uptake of curcumin and modulate the *in vivo* pharmacokinetics of curcumin due to albumin-binding interactions which are expected to enhance the efficacy of curcumin [203]. Curcumin also binds to a variety of biopolymers and is known to retain its medicinal activity in the bound states [204]. Kanai *et al.*, reported the first nanoparticle formulation of curcumin that demonstrates improved bioavailability in human subjects and C (max) for nanoparticle curcumin (named THERACURMIN) at 150 and 210 mg was 189 ± 48 and 275 ± 67 ng/mL (mean ± SEM), respectively in healthy human volunteers [205]. Enhanced bioavailability of curcumin in the near future is likely to bring this promising natural product to the forefront of therapeutic agents for treatment of human disease. Recent and ongoing clinical trials have indicated curcumin’s therapeutic potential against a wide range of human diseases with numerous signaling molecules targets. These preclinical studies have formed a solid basis for evaluating curcumin’s efficacy in clinical trials. In clinical trials, curcumin has been used either alone or in combination with other agents. A search on www.clinicaltrials.gov (accessed in February 2015) indicated that about 108 clinical trials with curcumin for the chronic diseases listed in this paper have been conducted, among which 31 clinical trials have been completed (Table 2). The most common evaluated human diseases for curcumin are cancer and inflammatory bowel disease. Most of these clinical trials are from the United States.

**Table 2:** Completed and on-going clinical studies of curcumin.

Diseases	Number of Clinical Studies		Mainly Completed Clinical Studies
	Completed	On-Going	
Neurodegenerative Diseases	3	2	1. A pilot study of curcumin and ginkgo for treating Alzheimer’s disease 2. Curcumin in patients with mild to moderate Alzheimer’s disease 3. A randomized, double-blind, placebo-controlled trial of curcumin in Leber’s hereditary optic neuropathy (LHON)
Diabetes	2	3	1. Effects of curcumin on postprandial blood glucose, and insulin in healthy subjects 2. Diabetes visual function supplement study
Obesity	0	1	1. Pilot study of curcumin for women with obesity and high risk for breast cancer
Cardiovascular Diseases	3	7	1. Curcumin (diferuloylmethane derivative) with or without bioperine in patients with multiple myeloma 2. Role of turmeric on oxidative modulation in ESRD patients 3. Diabetes visual function supplement study
Chronic Kidney Disease	2	2	1. Effect of oral supplementation with curcumin (turmeric) in patients with proteinuric chronic kidney disease 2. Role of turmeric on oxidative modulation in end-stage renal disease (ESRD) patients
Inflammatory Bowel Disease	5	14	1. Curcumin in pediatric inflammatory bowel disease 2. Curcumin + aminosalicylic acid (5ASA) *versus* 5ASA alone in the treatment of mild to moderate ulcerative colitis 3. Curcumin (tumeric) in the treatment of irritable bowel syndrome: A randomized-controlled trial 4. Curcumin biomarkers 5. Curcumin for the prevention of colon cancer
Allergy, asthma and bronchitis	1	2	1. Effect of supplemental oral curcumin in patientswith atopic asthma
Cancer	16	35	1. Curcumin (siferuloylmethane derivative) with or without bioperine in patients with multiple myeloma 2. A nutritional supplement capsule containing curcumin, green tea extract, *Polygonum cuspidatum* extract, and soybean extract in healthy participants 3. Curcumin for the prevention of radiation-induced dermatitis in breast cancer patients
Rheumatoid Arthritis	0	1	1. Curcumin in rheumatoid arthritis
Pancreatitis	0	1	1. Gemcitabine with curcumin for pancreatic cancer
Scleroderma	/	/	/
Psoriasis	1	1	1. Curcuminoids for the treatment of chronic psoriasis vulgaris

## 4. Conclusions

Curcumin has been demonstrated to have therapeutic potential for various chronic inflammatory diseases, essentially due to its anti-inflammatory and anti-oxidative properties against a vast array of molecular targets. Studies on the biological evaluation of curcumin have revealed that curcumin is a pro-drug, which inhibits the growth of cells by releasing active free thiol group within the target site. A large body of investigation has provided important insights into the anti-inflammation effects of curcumin which will constitute the basis for the further design and clinical application of extraordinarily potent drugs with potential therapeutic significance. As the problems of curcumin absorption, biodistribution, metabolism and elimination are overcome to enhance its bioavailability, many chronic inflammatory diseases will be at the forefront as promising targets for curcumin therapy.
